# New reimbursement model in Icelandic primary care in 2017: first-year comparison of public and private primary care

**DOI:** 10.1080/02813432.2022.2097713

**Published:** 2022-07-19

**Authors:** Hedinn Sigurdsson, Kristjan G. Gudmundsson, Sunna Gestsdottir

**Affiliations:** a Primary Care of the Captial Area, Gardabaer Primary Care Centre, Gardabaer, Iceland; b Reykjalundur Rehabilitation Centre, Mosfellsbaer, Iceland; c Department of Sport and Health Sciences, University of Iceland, Reykjavik, Iceland

**Keywords:** Primary care, healthcare financing, primary care choice reform, incentives, reimbursement model

## Abstract

**Objective:**

To analyze and compare the effect of a new reimbursement model (based on a modified version of the Swedish free choice reform) on private and public primary care in Iceland during its first year of use.

**Design:**

Descriptive comparison based on official data from the Ministry of Welfare, Directorate of Health, and the Icelandic Health Insurance on payments in the Icelandic primary care system.

**Setting:**

Primary care system operating in the Reykjavik capital area. Public primary care has dominated the Icelandic health sector. Both public and private primary care is financed by public taxation.

**Subjects:**

Fifteen public and four private primary care centers in the capital region.

**Main outcome measures:**

Different indexes used in the reimbursement model and public vs. private primary care costs.

**Results:**

No statistically significant cost differences were found between public and private primary care centers regarding total reimbursements, reimbursements per GP, number of registered patients, or per visit. Two indexes covered over 80% of reimbursements in the model.

**Conclusion:**

The cost for Icelandic taxpayers was equal in numerous indexes between public and private primary care centers. Only public centers got reimbursements for the care need index, which considers a patient's social needs, strengths, and weaknesses.

**KEY POINTS**
- The Icelandic primary care system underwent a reform in 2017 to improve availability and quality. A new reimbursement model was introduced, and two new private centers opened following a tender.
- Two out of 14 indexes cover over 80% of total reimbursements from the new model.
- Only 5 primary care centers, all publicly driven, got reimbursement for the care need index, which is a social deprivation index.
- Reimbursement systems should mirror the policies of health authorities and empower the workforce.

## Introduction

Strong primary care is grounded, among several other factors, on good access for the patients to derive correct information from the right health care professional at the right time [1]. In most of Europe, primary care is the first level of professional care; people present their health problems, and most of their curative and preventive health needs are satisfied [2]. General practitioners (GPs) are overwhelmed by packed schedules, inefficient work environments, and unrewarding administrative tasks. A recent systematic review [3] indicates that many GPs leave their current post due to higher professional titles, lower levels of income, lower job satisfaction, and lower morale. There seems to be better control over costs and better health outcomes in systems/countries with strong primary care [4]. The Alma Ata agreement between nations worldwide emphasizes strong primary care as a central feature in health care [5]. The European Union and the European Economic Area emphasize competition and tenders in the healthcare market, which also applies to primary care [6,7]. However, competition in a small market like the Icelandic one is controversial [8].

The Icelandic primary care system is state-funded. The system underwent a reform in 2017 to improve the availability and quality. Before the reform, the public sector dominated the Icelandic primary care system. The prototype for the new reform, the Swedish free choice reform, comes from Västra Götaland County in Sweden, which in 2007 allowed for the establishment of private primary care centers. The goal was to increase accessibility and responsiveness towards the needs of the patients, give patients a choice, and establish competition between centers. This change gave patients a choice between public, private for-profit, and private not-for-profit providers, all financed by regional taxation [9]. Lindström et al. [10] point out that as each county runs its own primary care, the reimbursement model for primary care in Sweden varies among county councils. For example, age is used as a primary index in 86% of counties, location of center in 66% of counties, and adjusted clinical group (ACG, explained in detail in Table 1) is used in 57% of counties, of which Västra Götaland is one. The models are determined mainly by capitation (an annual sum per listed individual adjusted for age, illness, and socioeconomic indicators), a fee-for-service (payment per visit), and a small portion of pay-for-performance according to specific set targets [11]. The Swedish free choice reform was inspired by reforms of the National Health Service (NHS) in the UK [12]. Following new reforms in Sweden, over 200 new primary care centers have been opened (although a few have since been closed), and only three companies run 20% of the market [13].

**Table 1.** List of indexes that represent reimbursement to primary care centers and proportional distribution of payments in 2017 and 2018 from each index.

Type of index	What the index stands for
^a^Cost index	Represents age, gender, and number of communications to a center.
2017: 41.1	An average individual has a cost index of 1.0.
2018: 40.9	
^a,b^ACG	ACG is based on two indexes: a weight index estimated with each person's disease burden in accordance with ICD-10 and a demand index that is standardized by dividing the weight index of each center with the average weight index from all centers.
2017: 41.1	
2018: 40.9	
^a,c^Care need index	CNI consists of the following seven demographical variables which vary in expense: 1) Proportion of individuals that are over age 65 and live alone; 2) Proportion of individuals who are recipient of disability benefits; 3) Proportion of individuals born abroad; 4) Proportion of unemployed individuals; 5) Proportion of single parents; 6) Proportion of new residents who are not Icelandic; 7) Proportion of children under age 5.
2017: 0.4	
2018: 0.4	
Quality index	How well centers serve a total of nine public health factors, e.g. indicators of diabetes; heart and pulmonary diseases; annual review of prescriptions for patients over age 70; smoking, blood pressure and BMI in risk groups; vaccinations; use of antibiotics.
2017: 4.8	
2018: 4.1	
Other indexes	Payments for service to primary schools, interpretation, psychologists, and prescriptions for exercise.
2017: 12.6	
2018: 13.6	

^a^Top two indexes explain about 82% of payments to centers both in 2017 and 2018; ^b^ACG: adjusted clinical group. ^c^Only 5 centers (all public) got paid for the Care Need Index [22].

Other Nordic countries have also been reforming their primary care systems. In Norway, changes to the primary care system were made in 2001 to enable GPs to manage their patient lists better and improve access to and integration with other services, such as specialized care [14]. In Finland, the latest changes in primary care starting in 2015 were inspired by the choice reform introduced in Sweden in 2007 [15]. In Iceland, the reimbursement model was introduced in 2017 in the Reykjavik capital area and in the rest of the country in January 2021. The main difference from the Swedish version is that the Icelandic government had restrictions about how many private practices could be opened. In the tender documents [16] from the Icelandic government, only three new primary care centers were allowed to be opened. It was also specified in the tender document where these new private centers should be stationed, contrary to the Swedish free-choice version. This resulted in two bids and only two new privately managed primary care centers opened in 2017 in Iceland following the tender offer .

The way in which different reimbursement models affect work motivation in private vs. public primary care is debatable, as financial incentives are widely used without fully understanding how they contribute to work motivation and staff income [17]. A study from Malta on patients' experiences in public and private primary care centers indicates that greater continuity of care and better doctor-patient relationships are found in the private sector [18]. No difference has been found concerning patients' experiences or public and private primary care efficiency in Iceland [19]. Even though the overall number of physicians per capita has increased in most European countries, the proportion of GPs has decreased and was, on average, 25% in Europe in 2016 [20]. In Iceland in 2016, 16% of all physicians were GPs, whereas this proportion was 15% in Sweden [20]. The utilization and distribution of resources within the health care system are constantly under review. Different approaches to funding the system have their positive and negative sides, and therefore other methods are used to correct reimbursement models if an unseen problem is found.

This study aims to analyze if differences are found between publicly and privately driven primary care centers regarding payments from the new reimbursement model. We hypothesize that no difference is observed in public- or private-driven primary care payments.

## Material and methods

This descriptive comparative study is based on official data from the Icelandic Health Insurance [21] on payments to primary care centers in the Reykjavik capital area from 2018. At that time, there were 223,894 individuals registered for the service of primary health care centers in the area. Some 22.3% (50,104) were serviced by the four private centers.

Before the reform, there were two private primary health centers in Reykjavík, one of which was on a fixed budget, like those run by the State. The other used capitation and payment per patient visit.

The fees are based on a new reimbursement model [22]. All payments reported in tables/figures and text are in Icelandic krona (ISK). In 2018, there were 15 public and 4 private primary care centers in the Reykjavik capital area, all financed by public taxes. Before the new reimbursement model, all primary care center funding was based on a fixed budget with discretionary changes.

The following description of the reimbursement model is based on the state budget and tender documents.

### The reimbursement model

The new reimbursement model assumes that patients receive basic services at their registered center. Thus, a center gets less reimbursement if a listed patient receives service elsewhere, for example at a primary care center at which the patient is not registered, from a specialist physician, at the emergency room at the hospital, or the emergency clinic (private-driven out-of-hours service run by GPs). Payments from the model go to the service provider. Reimbursement to each primary care center reflects the type of patients listed, and the service performed is estimated by five indexes explained in Table 1. Cost index and ACG produce the highest reimbursement (Table 1).

The average patient has a cost index of 1, while the elderly and young children have an index greater than 1 [21]. Care need index (CNI) (Table 1) and ACG are two risk-adjustment capitation methods used to increase accuracy in calculations of the care cost of patients. ACG uses diagnosis, age, and gender to estimate current cost, whereas CNI is used to estimate future care from a patient's education, area of residence, income, and other socioeconomic factors [23]. ICD-10 codes for chronic diseases give higher compensation than codes for minor problems.

Diagnosis codes assigned to patients are grouped by ACG methodology on the following five clinical and expected utilization criteria: (1) duration of the condition (acute, recurrent, or chronic); (2) severity of the condition (e.g. minor and stable versus major and unstable); (3) diagnostic certainty (symptoms focusing on diagnostic evaluation versus documented disease focusing on treatment services); 4) etiology of the condition (infectious, injury, or other); and 5) specialty care involvement (medical, surgical, obstetric, hematology, etc.). Unlike the Icelandic reimbursement model, which demands the use of ICD-10 and its three-digit codes, Västra Götaland's model uses a modified and simpler version of ICD-10 called *Klassifikation av sjukdomar och hälsoproblem 1997-Primärvård* (KSH97-P) (e. Classifications of diseases and health problems 1997 – Primary care) including codes with less than three digits. The principle is that all primary care centers are financed according to the same model, and the total budget for the model is determined annually.

The reimbursement system does not include payments for rent and home nursing care.

### Statistics

Official indicators from the aforementioned reports and other indicators (number of GPs at a center and number of registered patients) seen in Table 2 will be used to analyze the effect of the new reimbursement model on the primary care system in the Reykjavik capital area. In addition, t-tests were used to examine differences in reimbursements to public and private centers.

**Table 2.** Reimbursement to primary care centers in 2018, reported in thousands of Icelandic krona^a^ [21].

	Public		Private		
	*n* = 15		*n* = 4		*T*-test
	Mean	SD	Mean	SD	*P*-value
Cost index^b^	158,626^a^	31,214	157,599	52,071	0.975
Adjusted clinical group (ACG)	154,068	33,243	181,108	58,837	0.497
Quality index	4,645	1,267	5,293	1,284	0.471
Care need index (CNI)	2,074	3,696	0	0	–
Other indexes^c^	1,326	27,804	0,463	13,149	0.934
Total reimbursement	330,146	67,573	348,633	112,807	0.802
Reimbursement per GP	40,075	7,001	38,191	11,106	0.763
Reimbursement per registered patient	28,043	1,843	29,644	2,317	0.270
Reimbursement per visit	12,116	1,151	11,684	1,121	0.526

^a^To convert to euros, divide by ISK 139.4 which was the currency exchange rate on 1st December 2018, i.e. cost index of ISK 158,626,000 is then 1,137,919 euros; ^b^The index is standardized for different numbers of patients; ^c^School nursing, physical therapy, psychologists, interpreters, medication review, special income, payment to after-hour care.

## Results

**Figure 1.**
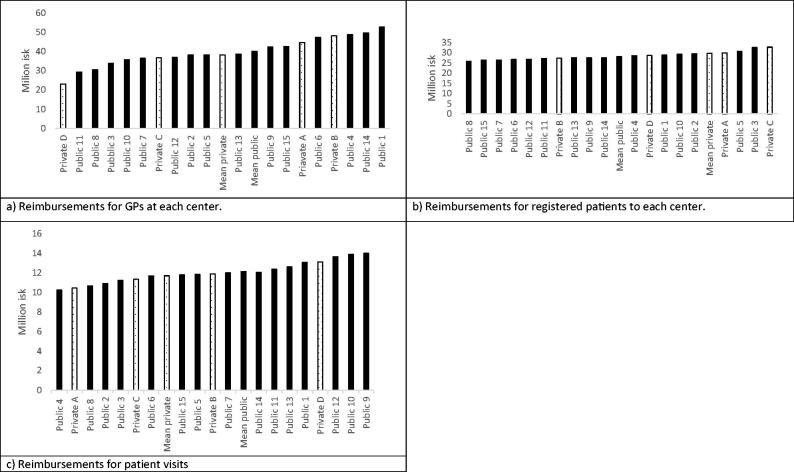
Reimbursements in 2018 for (a) GPs at each center, (b) all registered patients at a center, and (c) patient visits. The dark color is for public centers and dots represent private centers.

No significant difference was found in total reimbursements (reported in thousand Icelandic krona) to public (*M* = 330,146; SD = 69,945) vs. private centers (*M* = 348,634; SD = 130,259). Reimbursement per GP in 2018 was highest at a public center (52,578) and lowest at a private center (23,121) (Figure 1(a)). There was no significant difference between public (*M* = 40,075; SD = 7,000) and private (*M* = 38,191; SD = 11,106) centers in reimbursements per GP. In 2018, reimbursement per registered patient was highest at a private center (32,723) but lowest at a public center (25,739) (Figure 1(b)). There was no significant difference between public (*M* = 28,043; SD = 1,843) and private (*M* = 29,644; SD = 2,317) centers in reimbursements per registered patient. Reimbursement per patient visit in 2018 was highest and lowest at a public center (Figure 1(c)). There was no significant difference between public (*M* = 12,117; SD = 1,151) and private (*M* = 11,684; SD = 1,122) centers in reimbursements for patient visits in 2018.

**Figure 2.**
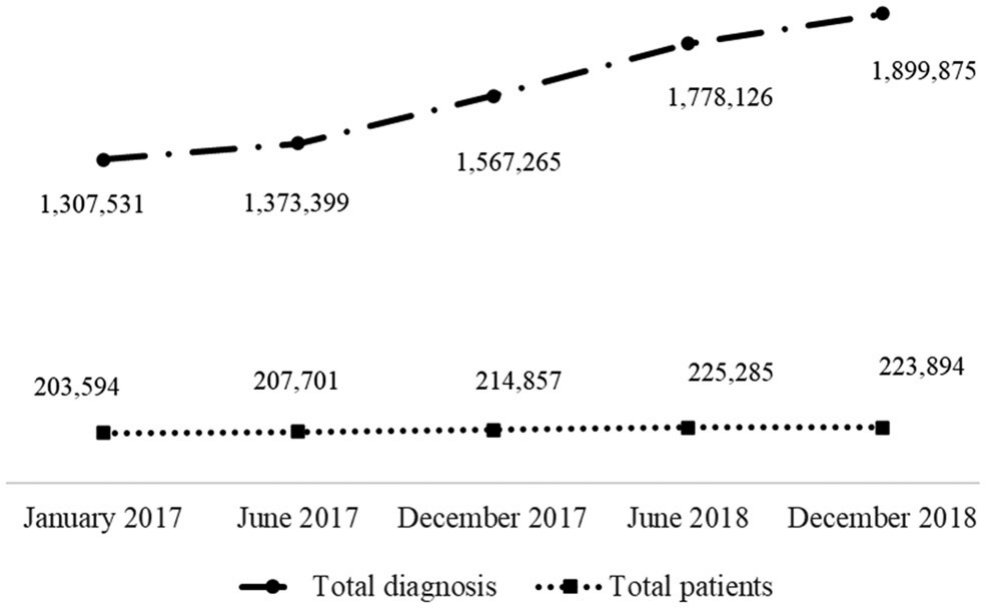
Increase in number of patients and diagnosis from 2017 to 2018 [Icelandic health insurance, personal communication, 27 January 2021].

The total number of visits each month to primary care centers increased by 10% from 2017 to 2018, and the number of registered patients increased by 4.3% during the same period. The increase in the total number of registered patients and number of diagnoses is seen in Figure 2. The estimated distribution of payments in the reimbursement model is shown in Table 1. The average reimbursement for the cost index for public centers was 158,624 and 157,599 for private centers. No significant difference was found between private and public centers in reimbursements for cost index. ACG was both highest and lowest at a private practice. No significant difference was found between private and public health care centers in reimbursement for ACG (Table 2). Cost index and ACG covered more than 80% of financing for each primary care center (Table 1). Only 0.4% of the funding in the model came from a CNI (Table 1), and only public centers got reimbursements for CNI.

## Discussion

No significant differences were found between public and private primary care centers regarding total reimbursements, reimbursement for cost index, ACG, payments per GP, payments per registered patient, or payments per visit. For ACG to determine almost half of all reimbursements in the Icelandic model is high. Because it is high, it can be speculated that ACG, compared with other indexes in the model, has lower validity due to subjective and new administrative evaluation of GPs on how to code diseases [24]. Only five centers, all public, got reimbursement for CNI. These five centers have demanding patients due to their socioeconomic status.

In 2014, four Swedish primary care centers received warnings by regulators as they registered diagnoses that were greater both in number and severity than there was support for. After what happened in Sweden, one is more doubtful about the ACG weighting in reimbursement models. However, a better measure of the extent of care and one that is impossible to manipulate is hard to find [25]. A Finnish study revealed that primary care staff was influenced by bonuses that followed improved recording of diagnoses [26]. The impact of payments on quality indicators has been found to be uncertain in general. There is no evidence that pay-for-performance provides better value for money than traditional pay schemes, and it can even be detrimental due to several ethical and practical concerns, including the risk of financial incentives being misused and patients being refused treatment [27].

A robust result from Sweden indicates that private primary care centers react to the incentives created by risk-adjustment of capitation [28]. The number of private primary care centers in Sweden has gone from 25% of all centers in 2007 to 40% in 2017 [29]. Iceland puts restrictions on private primary care centers by not allowing GPs to open new private centers. A weak association has been found between CNI and primary care visits per registered patient in Sweden which might reflect an insufficient compensation, lack of incentives or reimbursements being invested in other things than direct contact with patients [30]. After the new reform in Sweden in 2007, the primary care system still suffers from poor continuity and accessibility to GPs [10]. The reform in Sweden has been found to have had a negative impact on the provision of services for persons with complex needs [31]. The free market in Sweden might explain the greater prevalence of private providers in the affluent areas [32].

In the Directorate of Health's report [33] on primary care in the Reykjavik capital area, it is emphasized that quality indexes need to be revised to reflect better the service that primary care provides. It is also pointed out in the same report that the reimbursement model needs to be adjusted to the lack of GPs or lack of other staff at each primary center [33].

The new reform in Iceland has positively impacted better patient access to primary care centers and physicians' choice of work environments. The model is also a step forward as payments can be adjusted and focused on current public health issues. However, administration and surveillance are new items of expenditure. Although no significant differences were found in reimbursements for ACG and cost indexes between private and public centers during the study period, it could be speculated that it is not good that ACG represented almost half (40.2%) of payments from the new model. This can be assumed as a few years ago, the majority of county doctors in Sweden believed that ACG forces unjustified diagnoses [25], opening up the possibility of a moral hazard. It has been pointed out [34] that if a decision is made to distribute money *via* an index with a fixed total, fairness must be the guiding light.

The main strength of this study is that this is the first comparison of reimbursements to private and public primary care centers after a new reimbursement model was adopted in Icelandic primary care. Another strength of the study is that the data used are official data from the Icelandic Health Insurance. On the other hand, a weakness of the study is that it covers a one-year comparison with four privately driven centers, of which two started operating during the study period, compared to the 15 public-driven centers. In addition, there was an ongoing enlisting of patients to the new private centers during the study period, which might affect the results. Therefore, a more extended study period post-COVID pandemic would be of value.

Objective assessment of the performance of medical professionals is complex. The new reimbursement model is far from perfect, and an active consultation committee is working on adjusting the model as best as possible. However, reviewing and improving the model has not been a major focus since late 2019 due to demands following COVID-19.

As Nordic countries build their reimbursement health care system on a prototype from the USA, it is worth noting the recent changes in documentation requirements in the USA. These have been simplified considerably following the American Medical Association recommendations of alignment of financial incentives, all of which are aimed at achieving better patient outcomes by reducing inefficient administrative documentation, especially if this can save a typical office-based GP many hours per year in documentation [35].

## Conclusion

No differences were found between private and public primary care regarding reimbursements during the study period. The cost for Icelandic taxpayers was equal in numerous indexes between public and private primary care centers. CNI was only paid to public primary health care centers. With the reform, competition for patients has been introduced as reimbursement to centers follows the number of patients listed, which will hopefully improve patient service. Future research is needed to answer how the new reimbursement system affects physicians and managers at primary care centers. Such research could be carried out using semi-structured interviews. Reimbursement systems should mirror the policies of health authorities that have the patients' needs in mind as well as empowering the workforce.
